# Automated Tumour Recognition and Digital Pathology Scoring Unravels New Role for PD-L1 in Predicting Good Outcome in ER-/HER2+ Breast Cancer

**DOI:** 10.1155/2018/2937012

**Published:** 2018-12-17

**Authors:** Matthew P. Humphries, Sean Hynes, Victoria Bingham, Delphine Cougot, Jacqueline James, Farah Patel-Socha, Eileen E. Parkes, Jaine K. Blayney, Michael A. O'Rorke, Gareth W. Irwin, Darragh G. McArt, Richard D. Kennedy, Paul B. Mullan, Stephen McQuaid, Manuel Salto-Tellez, Niamh E. Buckley

**Affiliations:** ^1^Centre for Cancer Research and Cell Biology, Queen's University Belfast, Belfast, UK; ^2^Horizon Discovery Ltd, 8100 Cambridge Research Park, Waterbeach, Cambridge, CB25 9TL, UK; ^3^College of Public Health, The University of Iowa, Iowa City, IA 52242, USA; ^4^School of Pharmacy, Queen's University Belfast, Belfast, UK

## Abstract

The role of PD-L1 as a prognostic and predictive biomarker is an area of great interest. However, there is a lack of consensus on how to deliver PD-L1 as a clinical biomarker. At the heart of this conundrum is the subjective scoring of PD-L1 IHC in most studies to date. Current standard scoring systems involve separation of epithelial and inflammatory cells and find clinical significance in different percentages of expression, e.g., above or below 1%. Clearly, an objective, reproducible and accurate approach to PD-L1 scoring would bring a degree of necessary consistency to this landscape. Using a systematic comparison of technologies and the application of QuPath, a digital pathology platform, we show that high PD-L1 expression is associated with improved clinical outcome in Triple Negative breast cancer in the context of standard of care (SoC) chemotherapy, consistent with previous findings. In addition, we demonstrate for the first time that high PD-L1 expression is also associated with better outcome in ER- disease as a whole including HER2+ breast cancer. We demonstrate the influence of antibody choice on quantification and clinical impact with the Ventana antibody (SP142) providing the most robust assay in our hands. Through sampling different regions of the tumour, we show that tumour rich regions display the greatest range of PD-L1 expression and this has the most clinical significance compared to stroma and lymphoid rich areas. Furthermore, we observe that both inflammatory and epithelial PD-L1 expression are associated with improved survival in the context of chemotherapy. Moreover, as seen with PD-L1 inhibitor studies, a low threshold of PD-L1 expression stratifies patient outcome. This emphasises the importance of using digital pathology and precise biomarker quantitation to achieve accurate and reproducible scores that can discriminate low PD-L1 expression.

## 1. Introduction

DNA-based high-throughput mutational analysis [1, 2] and high-throughput gene expression profiling [3] have demonstrated that breast cancer is a heterogeneous disease and treatment options must reflect this. Despite major breakthroughs such as the targeted therapies against the estrogen receptor (ER) or oncogenic proteins such as HER2, 20% of patients will relapse with secondary breast cancer, which is currently incurable. This may be due to the underlying inter- and intratumoural heterogeneity leading to intrinsic and/or acquired resistance to therapy, or to basic molecular pathways (such as angiogenesis) not controlled by these therapies.

It has long been thought that the ultimate cure for cancer would arise from harnessing the host immune system that, through its adaptive nature, could eradicate cancer cells even as they evolve. Indeed, in breast cancer, the presence of Tumour Infiltrating Lymphocytes (TILs) is a positive prognostic factor [4, 5] and a number of immune based gene expression signatures identify good outcome subgroups especially within the poor outcome triple negative subgroup [6–9]. Central to this is the theory of immunosurveillance, dating from the 1950s [10] and the subsequent concept of immunoediting, providing a major selective pressure for cancer cells to acquire ways of evading and/or neutralising the immune cells [10]. This is borne out in the two main immune phenotypes observed in a range of solid tumours [11], namely, T-cell rich and T-cell poor (the latter associated with denser stroma and alternative macrophage populations). The T-cell rich tumours are associated with an inflamed microenvironment and high expression of immune inhibitory pathways that allow the tumour to evade destruction by these cells. One such pathway is the PD1/PD-L1 immune checkpoint pathway. Expression of PD-L1 is observed in multiple cancer subtypes including breast, suggesting that cancer cells have hijacked this mechanism in order to evade immune mediated destruction [12, 13]. This observation is the basis for drug targeting by immune checkpoint inhibitors allowing reactivation of the immune system [14]. Clinical trials in multiple cancer types have shown some remarkably durable responses as reviewed by [15].

Most predictors of drug response also have a pure prognostic value. Indeed, PD-L1 expression has been studied in multiple cancer types and correlated with various clinicopathological parameters [16–19]. In general, studies to date have used different monoclonal antibodies for immunohistochemistry (IHC) and different techniques beyond IHC [20–24], with different scoring systems and in different clinical trial materials (reviewed in [25–27]) providing inconsistent findings. This is also the case in breast cancer. Whereas early studies demonstrated that PD-L1 expression was associated with other poor prognostic factors [28–30], later studies have qualified this observation in triple negative (TNBC) and/or basal-like breast cancer [20, 24, 31, 32]. Subject to the same technical inconsistencies, PD-L1 itself has been shown to be both a positive and negative prognostic factor, although most studies indicate that high PD-L1 expression is associated with better clinical outcome as well as increased TILs [21, 23, 24]. Furthermore, high PD-L1 expression has been reported to be a predictive biomarker of neo-adjuvant chemotherapy [20, 23, 33].

At the heart of this conundrum is the subjective scoring of PD-L1 IHC. Current standard scoring systems for PD-L1 as a companion diagnostic involve separation of epithelial and inflammatory cells and find clinical significance in percentages of expression above or below 1% [34, 35]. Clearly, an objective, reproducible and accurate approach to PD-L1 assessment would bring a degree of necessary consistency to this landscape. Therefore, we present a systematic comparison of technologies and scoring systems and the application of QuPath, a digital pathology platform [36], to the scoring of PD-L1 in breast cancer, starting with TNBC as a paradigm, and then extending its application to breast cancer as a whole. We show how QuPath's analytical tools (automated tumour recognition, automated separation of epithelial and immune compartments, and dynamic threshold identification) confirm many of the observations stated before and add further insight to the value of PD-L1 expression in breast cancer subgroups.

## 2. Materials and Methods

### 2.1. Study Design and Patient Selection

We conducted a retrospective case-control study involving a cohort of TNBC patients; ethical approval was obtained and tissue was acquired through the Northern Ireland Biobank (NIB ref: 15-0168). The dataset was compiled for a cohort of 109 patients and included a range of clinical and pathological parameters (Supp Table 1a). All of the resection samples were processed and reported in the Belfast Health and Social Care Trust. Surgical resection of tumour was performed between 2000 and 2013 with a median follow-up of four years. All breast cancer resection samples were placed into 10% buffered formalin for 48-72 hours to allow adequate sample fixation. All samples were from Cellular Pathology Laboratory, Belfast Health and Social Care Trust and were fixed and processed to paraffin using UKAS accredited standard protocols. Paraffin-embedded blocks were stored in a cool, sunlight free environment. Original hormone receptor and HER2 scores were available to define the TNBC cohort. This was then confirmed following construction of the tissue microarray (TMA). Patient exclusion criteria included male sex and past history of cancer of any type. Additional cohorts used in this study have been previously described in detail [37] with a summary provided (Supp Table 1b).

### 2.2. TMA Construction

Original haematoxylin and eosin (H&E)-stained sections of all of the blocks were reviewed for tumour block selection. Following block selection, a new H&E section was cut and the slide was annotated for TMA construction. Where possible, from each selected donor block, nine representative areas were annotated for targeted coring – 3 tumour epithelial rich cores (T), 3 tumour cores from lymphoid rich areas (TL), and 3 stromal rich tumour cores (TS) in the 90-patient cohort. The TMA was constructed with 1-mm-diameter cores using a manual tissue arrayer (Beecher Instruments, Silver Spring, MD, USA) as described previously [38].

### 2.3. Horizon PD-L1 Reference Standard Panel

The PD-L1 IHC Reference Standard consists of a genetically defined 11-spot custom cell line array (CLMA) with a range of negative, low, medium, and strong protein expressing cell lines developed using Horizon Discovery's gene editing technology. Parental cells were purchased from a commercial source and were characterised short tandem repeat (STR) analysis. Cell lines were engineered under the control of promoters of different strengths that resulted in a range of controlled PD-L1 protein expressing cell lines by IHC. Individual cell lines in the 11 spot CLMA were extensively characterized and verified using molecular assays, IHC, and quantitative digital pathology by Horizon Discovery.

### 2.4. Immunohistochemistry

All IHC was performed in an integrated laboratory (Northern Ireland Molecular Pathology Laboratory) that has UK Clinical Pathology Accreditation [39]. Sections were cut from the whole face or TMA blocks for H&E staining and IHC. The initial H&E section was used to assess TMA quality and appropriate tumour, lymphoid, or stromal content for subsequent IHC localizations and analysis. Sections for IHC were cut at 4 microns on a rotary microtome, dried at 37°C overnight, and then used for IHC that was performed on automated immunostainers (Ventana BenchMark or Leica Bond-Max, Milton Keynes, UK). See Supp Table 2 for primary antibody, pretreatment, and biomarker detection details all of which were optimised prior to application to TMA sections. Subsequently, all sections were visualized with diaminobenzidine, counterstained with haematoxylin, and then dehydrated and tape-mounted using a Sakura autostainer. All slides were scanned on an Aperio AT2 Digital scanner at x40.

### 2.5. Tumour Infiltrating Lymphocyte (TIL) Assessment

TILs were assessed by three independent experienced immunohistochemists on H&E sections as recommended by the International TILs working group 2014 [40]. TILs were reported as percentage stromal TILs of the whole face sections or Tumour rich (T) cores of the TNBC TMA.

### 2.6. Image Analysis

Digital pathological analysis of the IHC stained TMA slides was performed using QuPath [36, 41], an open-source image analysis programme created in-house. All x40 scanned slides were imported into the programme. Dearraying of the TMA and tissue detection was carried out to identify the areas of tissue available for cellular analysis. Cores were removed following strict exclusion criteria; e.g., tissue cores that contained <100 tumour cells were removed from analysis. Rigorous QC steps were taken to remove necrosis, tissue folds, and entrapped normal structures; this was confirmed by a second reviewer with frequent consultation. To minimise the risk of false positive detection, manual removal of positive macrophage staining was conducted as PD-L1 expression by macrophages is well recognised and edited out where possible. Positive staining was defined as the presence of any discernible DAB positivity localised in the membrane and/or cytoplasm. Instances of punctate granular DAB staining were considered positive and in highly expressing cases a strong expression pattern of membrane staining was seen on tumour epithelial cells.

Quantitation of PD-L1 was conducted as previously described [36]. Briefly, intensity thresholds were set for cellular DAB detection, under consultation with a pathologist. Percentage positive data was extracted from each TMA core and averaged across replicates.

### 2.7. RNA In Situ Hybridization for PD-L1

Manual chromogenic RNAScope for PD-L1 (Hs-CD274 transcript variant 1: sequence region 124-1122 cat. No. 600861, Advanced Cell Diagnostics, 3960 Point Eden Way Hayward, CA 94545, USA) was performed on TMA sections as previously described [42]. RNAscope for positive control probe PPIB (313910 Accession # NM_000942.4) and negative control probe DapB (310043 Accession # EF191515) were also performed on selected TMA sections. Image analysis on selected regions of interest within the TMA cores of PD-L1-probe was performed using Spotstudio Software from ACD with user-defined thresholds after slides were scanned using an Aperio scanner at x40 resolution.

### 2.8. Statistical Analysis

Lin's concordance analysis was used to examine the relationship between the PD-L1 scores using different antibodies with two-sided correlation coefficients reported. Results were interpreted using the cutoffs proposed by McBride [43]. Fisher's exact test was used to test correlation between PD-L1 and lymph node status. Chi-squared test was used to assess correlation between PD-L1 and CD4 or CD8. Numbers were converted to percentages for graphing purposes only. A Mann Whitney test was used to test the association between PD-L1 and immune gene expression and TIL scores. Kaplan Meier curves were used to investigate the relationship between PD-L1 and patient survival using the log-rank test or alternatively Cochran-Mantel-Haenszel chi-squared test where indicated using Prism6. Multivariate analysis and step-wise regression were carried out using the R package MASS using the clinical parameters Age, Tumour Stage (categorised as T1 and T2-4), and Lymph node involvement. All Hazard ratios and p-values are presented in Supp Tables 4-6. Statistical tests were two sided, and p-values less than 0.05 were considered to be statistically significant.

## 3. Results

**Figure 1:**
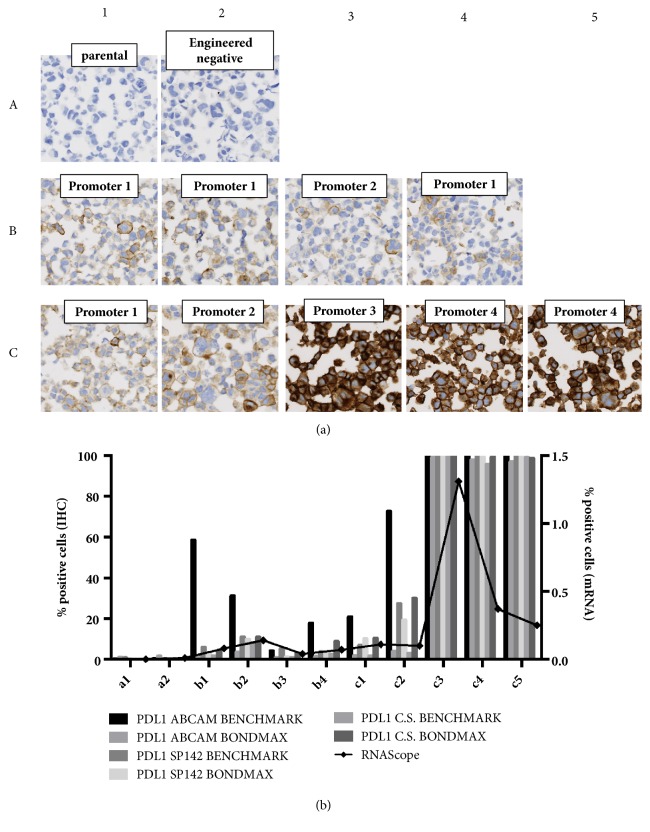
(a) Map of the Horizon Discovery PDL1 Reference Standard panel demonstrating negative, low, medium, and strong protein expression (Data provided by Horizon Discovery). (b) Histogram of IHC-determined protein and RNAScope-determined mRNA expression of PDL1 in the Horizon Discovery custom cell line panel. IHC was performed with 3 different antibodies (Abcam (28-8), Spring Bioscience (SP142), and Cell Signalling (405.9A11)) and on the Bondmax and Benchmark automated platforms. IHC was quantified using the QuPath software. RNAScope was quantified using SpotStudio (ACD).

In order to assess the reliability of PD-L1 analysis using the digital pathology platform, QuPath, we first utilised a genetically defined 11-spot custom cell line array PD-L1 IHC Reference Standard from Horizon Discovery (Figure 1(a)). We compared three commercially available antibodies [22, 44–47] across different automated staining platforms. Analysis showed almost no PD-L1 staining in the null cell line standards (A1-2) and similar high expression in the high expressing cell line standards (C3-6). More varied expression was observed in the low to medium expressing cell line standards (B1-4, C1-2). In general there was good concordance between the different staining protocols with the exception of the Abcam (28-8) antibody using the BenchMark automated staining platform (Supp Table 3). When the technical reproducibility of each antibody was compared based on platform choice, excellent concordance was observed for SP142 with similar levels of expression detected regardless of platform. More disparate levels of expression that varied according to platform choice were observed for the Cell Signalling and Abcam antibodies (Figure 1(b)). SP142 was therefore chosen for further IHC studies given the observed technical reliability and its clinical relevance as a companion diagnostic [48].

In addition to IHC, PD-L1 may also be quantified using in situ hybridisation using RNAScope. In situ PD-L1 mRNA expression was not detectable in the null cell line standards (A1-2). As seen with the IHC experiment, more varied expression was observed for the low to medium expressing cell line standards (B1-4, C1-2). Increased expression was seen in the high cell line standards (C3-5) although there appears to be discordance between protein expression (measured by IHC) and mRNA (as measured by RNAScope) in 2 of the 3 high cell lines (C4-5) (Figure 1(b)). This could reflect posttranslational regulation of PD-L1 protein or the fact that the protein based quantification does not account for intensity of staining.

Based on these results, we took forward the SP142 IHC on the Benchmark platform as well as the RNAScope assay for assessment in patient samples.

**Figure 2:**
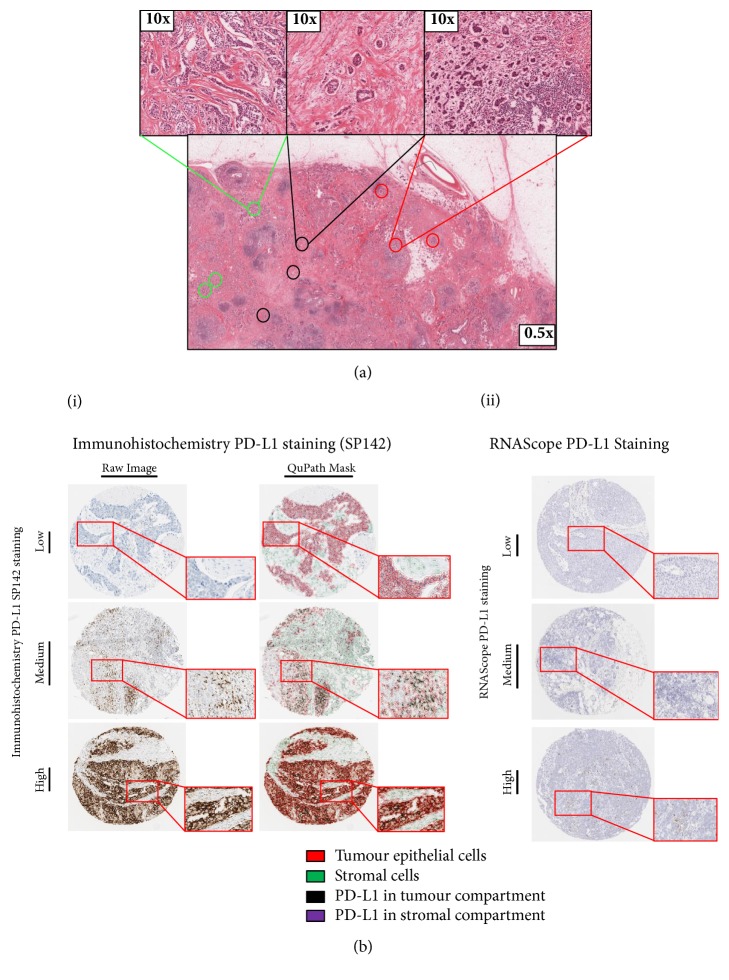
(a) Areas of tumour cores targeted for use in subsequent tissue microarrays. Where sufficient material was available, three cores of tissue were taken from three areas, (1) enriched with tumour epithelium (green), (2) enriched with tumour stroma, (<50% tumour epithelium) (black), and (3) enriched for lymphoid infiltrates within the tumour (red). (b) (i) Representative cores ranging from absent to high PD-L1 protein expression assessed by IHC using SP142, as indicated. Both raw immunohistochemistry images and QuPath cell detection masks are shown. A magnified region is shown in an exploded view. The key displays the QuPath cell classifier on the mask images. (ii) Displays PD-L1 expression identified by RNAscope. Cores ranging from absent to high PD-L1 RNAScope expression, as indicated. A magnified region is shown in an exploded view.

In order to fully investigate the role of PD-L1 as a biomarker we initially focussed our assessment on TNBC as it demonstrates the greatest range of PD-L1 expression and most significant correlation with patient outcome. Therefore, we constructed a bespoke TMA that would allow us to compare not only the different technology platforms (IHC, RNAScope) but also different tumour areas. For each patient a total of nine cores were selected; three tumour rich cores (T), three tumour cores with high stroma (TS), and three tumour cores tumour from a lymphoid rich region (TL) (Figure 2(a) and Supp Fig1A).

Microscopic IHC analysis with SP142 PD-L1 antibody on individual TMA cores demonstrated various expression patterns of PD-L1 in the different tissue compartments (Figure 2(b)(i)). When present on tumour epithelial cells the pattern was of striking membranous staining coupled with a granular cytoplasmic staining. Within the lymphoid compartment, or in individual tumour infiltrating lymphoid cells, punctate cytoplasmic staining was observed with occasional cells showing a more intense perinuclear pattern. As to be expected using in situ mRNA detection, RNAScope based assessment of PD-L1 expression showed varying numbers of dots per cells within both tumour epithelial and lymphoid cell types (Figure 2(b)(ii)). All tissues were deemed adequate for RNAscope assay by expression of the positive control probe PPIB. No expression of the negative control probe bacterial DapB was observed in any of the tissue samples.

**Figure 3:**
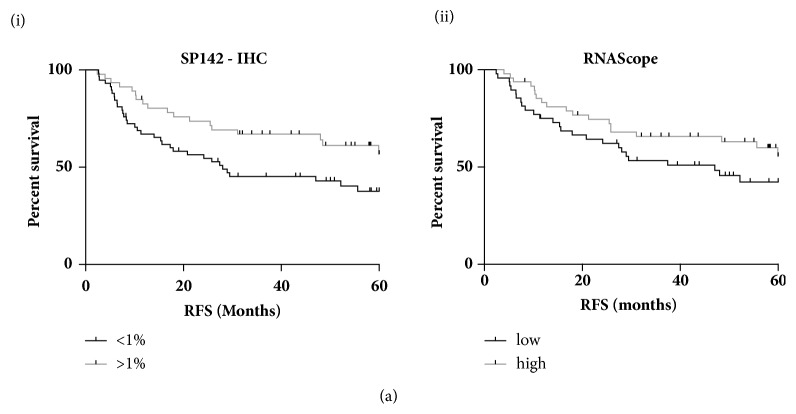
Kaplan Meier Plots of relapse free survival stratified based on PD-L1 expression above or below 1% as determined by the (i) SP142 or (ii) by RNAScope.

In order to compare the quantification of PD-L1 by the different technologies and the associated correlation with clinical outcome, initial automated analysis was based on total (epithelial and lymphoid) PD-L1 expression across all 9 cores per patient. A threshold of 1% was used to stratify IHC-based PD-L1 expression as high (>1%) or low (<1%) in keeping with the clinically relevant thresholds reported in the literature with 44% of cases called PD-L1 positive using the SP142 antibody. Median expression was used to dichotomise the mRNA expression. While similar trends were observed with high PD-L1 expression associated with increased relapse free survival (RFS), only assessment of PD-L1 expression by IHC using the SP142 antibody was significant correlation (HR0.536 (95%CI 0.316-0.94) p=0.0294) (Figure 3). Similar results were also seen with overall survival (OS) though this did not reach significance (Supp Fig1B). While the assessment of 9 cores representing three different regions of the tumour provides some insight into intratumoural heterogeneity, seventeen whole face sections (representing low, intermediate, and high PD-L1 expression) were also assessed by IHC (Supp Fig1C). All cases that scored negative in the TMAs were confirmed as PD-L1 negative in the whole-face sections. All PD-L1 positive cases in the TMA settings were confirmed as positive in the whole-face setting. While variation in the percentage of tumour epithelial cells expressing PD-L1 was observed, there was significant correlation between whole face and TMA-derived PD-L1 expression (p=0.0019). Based on these findings an IHC-based approach to quantify PD-L1 using the SP142 antibody was applied in all further experiments.

**Figure 4:**
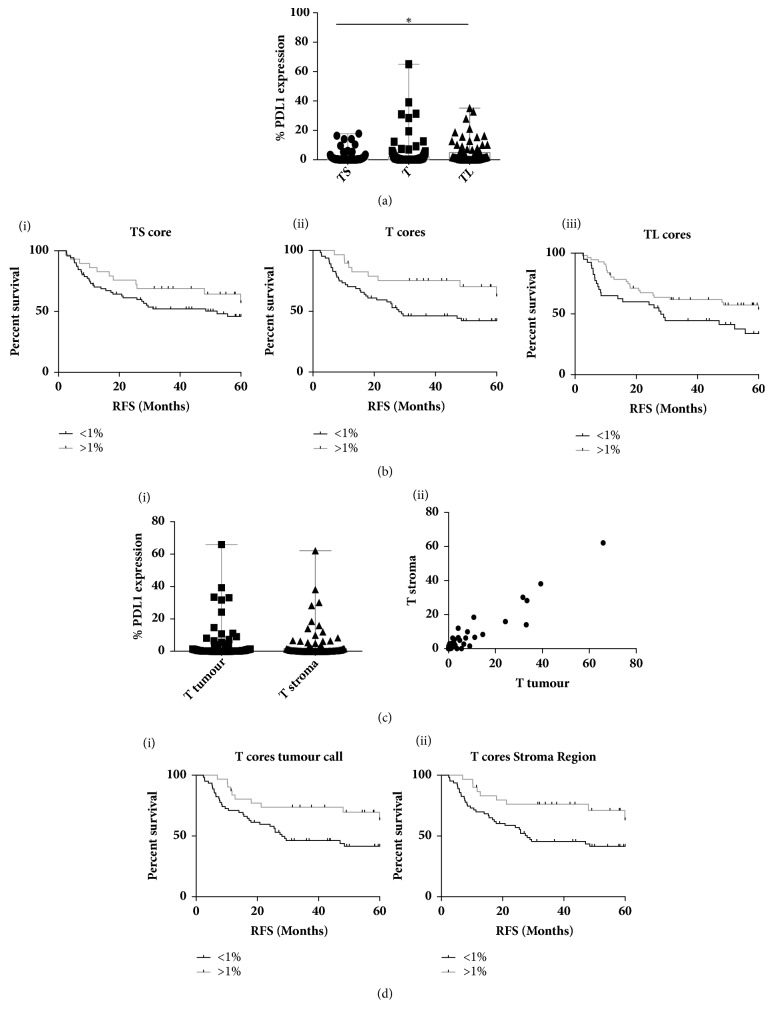
(a) Box and whisker plot showing the range min. to max. of PD-L1 expression (determined by SP142) in the three different tumour core types: stroma rich (TS), tumour rich (T), and lymphoid rich (TL). (b) Kaplan Meier Plots of relapse free survival stratified based on PD-L1 expression above or below 1% in the three different core types (i)TS, (ii)T, and (iii) TL. (c) (i) Box and whisker plot showing the range of min. to max. and (ii) the correlation between tumour and stroma derived PD-L1 expression within the tumour rich (T) cores. (d) Kaplan Meier Plots of relapse free survival stratified based on (i) tumour or (ii) stroma derived PD-L1 expression above or below 1%.

Next we wanted to compare the correlation with survival of PD-L1 expression in the different regions of the tumour (TS, T and TL). Galon and colleagues were the first to highlight the importance of the immune contexture showing that type, density, and location of immune cells within colon cancer predict clinical outcome [49, 50]. The expression of PD-L1 varied significantly between the tumour regions (p=0.032) with the greatest range of PD-L1 expression observed in the T cores (range 0–65.05%) (Figure 4(a)). This appears to be of clinical importance while high PD-L1 expression was associated with improved outcome in all core types; only the tumour rich (T) cores showed significance in terms of RFS (HR 0.445 (95%CI 0.271-0.917) p= 0.0255) (Figure 4(b)) with the same trend observed for OS though not quite reaching significance potentially due to the length of follow-up data available (5yr)(Supp Fig2A).

Following on from this we wanted to further determine the importance of the cell context of PD-L1 expression. Therefore PD-L1 was categorised based on expression within the stroma (lymphoid cells) vs the tumour nest (tumour epithelial and lymphoid cells). Analysis was restricted to the tumour rich (T) cores given the significant correlation with clinical outcome. The range of expression was very similar and there was a highly significant correlation between stromal and tumour derived expression (R=0.9569, p= <0.0001) (Figure 4(c)). Furthermore, only 5 patients with >1% stromal expression had <1% tumour expression and conversely only 6 patients displayed >1% tumour expression with <1% stromal expression. Given this strong overlap, it was not surprising that both high tumour and stromal derived PD-L1 were significantly associated with improved RFS (Tumour HR 0.455 (95%CI 0.274-0.909) p=0.0237, Stroma HR 0.421 (95%CI 0.26-0.872) p=0.0164) (Figure 4(d)) with similar though nonsignificant trends for OS (Supp Fig2B).

**Figure 5:**
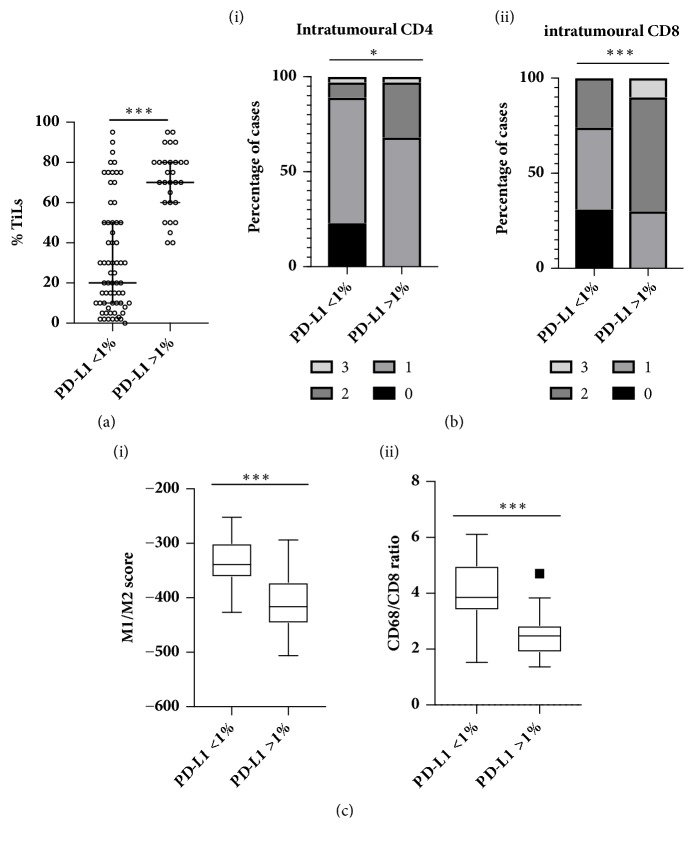
(a) Scatter plot of percentage stoma TILs (median and interquartile range indicated) in cases determined to be low (<1%) or high (>1%) for PD-L1 expression (whole core assessment) in the tumour rich cores. (b) Intratumoural (i) CD4 and (ii) CD8 expression in PD-L1 low (<1%) and high (>1%) samples. (c) Box and whisker plots of gene expression derived (i) M1/M2 signature scores and (ii) CD68/CD8 ratio in PD-L1 low (<1%) and high (>1%) samples.

We next wanted to investigate the correlation between PD-L1 and the immune microenvironment. First, we assessed Tumour Infiltrating Lymphocytes (TILs) using the methodology recommended by the International TILs Working Group 2014 [5] to report percentage stromal TILs. Using the same cases utilised to assess PD-L1 whole face expression, a highly significant correlation between percentage TILs assessed in TMA cores and whole face sections was observed (p = <0.0001, R^2^ = 0.7456). Furthermore, as previously reported in the context of anthracycline based chemotherapy [5, 51], high TIL expression was significantly correlated with improved survival when analysed as a continuous variable per 10% increase (RFS HR 0.8611 (95%CI 0.7855-0.944) p=0.00143, OS HR 0.8723 (95%CI 0.7811-0.9741) p=0.0153) or when a receiver operating characteristic (ROC) curve was used to determine the best cut-off and patient dichotomised based on low (<30%) or high (>30%) stromal TILs (SuppFig2C). Of interest, this cut-off was shown to have clinical relevance in assessing the prognostic and predictive value of TILs in HER2+ breast cancer [52]. A weak but highly significant correlation was observed between PD-L1 expression and percentage TILs in T cores (p=0.0001, R^2^ = 0.151) and when analysed in the context of low (<1%) and high (>1%) PD-L1, the percentage of TILs observed was significantly higher in the high PD-L1 cases (p = <0.0001) (Figure 5(a)). It is important to note that a number of PD-L1 low cases expressed high levels of TILs indicating distinct biology driving these immune-related biomarkers.

In order to further characterise the composition of the immune infiltrate in the context of PD-L1 expression, we utilised IHC and gene expression analysis that had been previously carried out on a subset (67/109) of the TNBC cohort [9]. High PD-L1 expression was significantly associated with high intratumoural CD4 (p=0.0123) and CD8 (p=0.0004) (Figure 5(b)). No correlation was observed for stromal CD4 or CD8 or FOXP3 (intratumoural or stromal). Furthermore, high PD-L1 was also associated with a more “anti-tumour” M1-like phenotype as evidenced by the significantly higher gene expression signature score (p=<0.0001) [53] and a significantly lower ratio of CD68/CD8 gene expression (p= <0.0001) [54] (Figure 5(c)).

**Figure 6:**
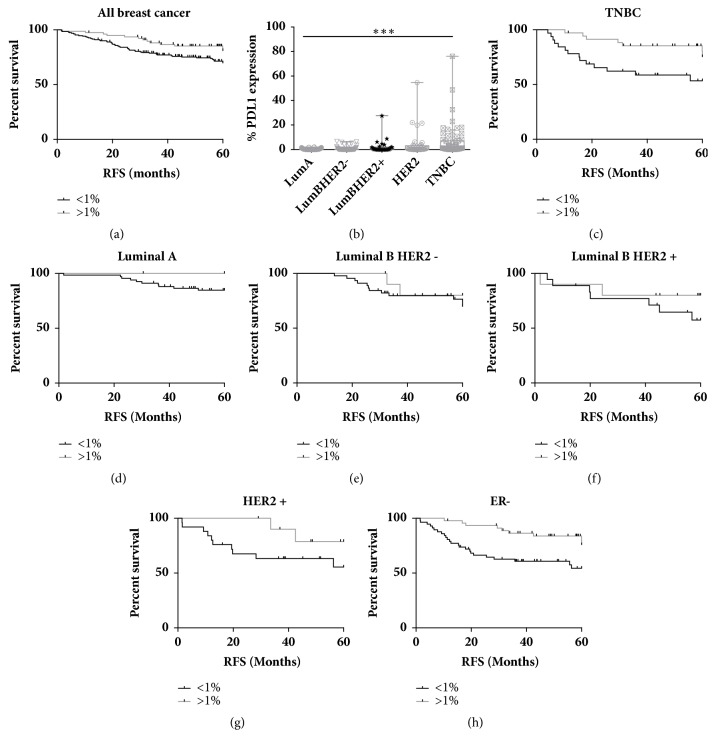
(a) Kaplan Meier Plots of relapse free survival stratified based on PD-L1 expression above or below 1% in breast cancer as a whole. (b) Box and whisker plot showing the range (min. to max.) of PD-L1 expression in the different subtypes of breast cancer. Kaplan Meier Plots of relapse free survival stratified based on PD-L1 expression above or below 1% in (c) TNBC, (d) Luminal A, (e) Luminal B/HER2 negative, (f) Luminal B/HER2 positive, (g) HER2 positive, and (h) ER negative breast cancer.

We then wanted to extend the analysis of PD-L1 expression into other breast subtypes and this was achieved using an additional cohort accessed through the Northern Ireland Biobank (NIB). This was made up of 300 breast cancer patients, all of which were treated with FEC, creating a relatively uniform clinical cohort [37]. Given the fact that, during the routine annotation and construction of TMAs within the NIB, epithelial rich regions are selected and therefore are comparable with the tumour rich (T) cores from the bespoke TNBC TMA, we felt that this TMA was suitable for analysis. In addition, most TNBC cases within the 300 cohort (n=59/69) are represented on the bespoke TNBC TMA allowing direct comparison. Based on our previous results, we did not classify PD-L1 expression based on tumour or stromal expression but instead determined expression of the entire core. 29% of cases were determined to be PD-L1 positive (>1%) and interestingly, this was shown to be significantly associated with improved RFS when the cohort was analysed as a whole (HR 0.547 (95%CI 0.35-0.994) p= 0.0477) (Figure 6(a)). We then decided to look at PD-L1 expression and its potential correlation with outcome within the subgroups as defined by the St Gallen Classification [55]. There was a highly significant variation (p<0.0001) in expression across the different subtypes with the greatest range (0-76.04%) of PD-L1 expression observed in the TNBC cases which is consistent with previously published data (Figure 6(b)). Of interest, HER2 enriched samples also displayed a broad range of expressions albeit not as great as TNBC.

As expected, high PD-L1 expression was associated with improved RFS and OS in TNBC patients (HR 0.355 (95%CI 0.148-0.84) p= 0.019 and HR 0.255 (95% CI 0.087-0.861) p=0.0269, respectively) (Figure 6(c) and Supp Fig3B). However, analysis of the other breast cancer subgroups revealed some novel findings. High PD-L1 expression was associated with improved RFS in Luminal A patients through given the low number of patients with high expression (<13%); this failed to reach significance (Figure 6(d)). Furthermore, this trend was not seen in overall survival (Supp Fig3C). PD-L1 expression was not associated with clinical outcome in the highly proliferative Luminal B samples regardless of HER2 status (Figures 6(e) and 6(f) and Supp Fig3D&E). Unexpectedly, high PD-L1 was associated with improved survival in the HER2 enriched subgroup. While this was not significant in terms of RFS (Figure 6(g)), it did reach significance in OS with no deaths observed in patients with high PD-L1 (HR 0.215 (95%CI 0.062-0.745) p=0.0153) (Supp Fig3F). This leads us to investigate the prognostic role of PD-L1 in ER negative disease as a whole with the novel finding that high PD-L1 is significantly associated with improved RFS and OS in this subset of breast cancer (HR 0.36 (95%CI 0.195-0.761) p=0.0063 and 0.154 (95%CI 0.103-0.526) p=0.0005, respectively) (Figure 6(h) and Supp Fig3G). This remained significant at a multivariate level with a stepwise regression model demonstrating that both PD-L1 expression and lymph node involvement are both significantly associated with clinical outcome (Supp Table 6C&D). Further analysis showed no correlation between these two variables.

This is an important finding for this poor outcome group of patients which represents 20-30% of all breast cancer cases and is associated with a disproportionately high number of deaths especially in the first five years following diagnosis.

## 4. Discussion

The role of PD-L1 as a prognostic and predictive biomarker is an area of great interest within oncology due to its association with the novel immune checkpoint inhibitors. It is also showing great promise in the treatment of a range of cancer types. However, inconsistent results have been obtained in the analysis of the PD-L1 biomarker. We propose that lack of an objective, reproducible and accurate approach to PD-L1 quantification and scoring may, at least in part, underlie this. Therefore, the aim of this study was to perform a comprehensive study of PD-L1 as a biomarker in breast cancer using automated tumour recognition and quantitative IHC image analysis together with objective scoring systems on uniform patient tissue cohorts with extensive clinical annotation. We show that the use of different antibodies during IHC can have a significant impact on the detected PD-L1 expression and its association with clinical outcome. This was compared to mRNA expression as determined by RNAScope. We show here that high PD-L1 expression, as measured by IHC using the Ventana SP142 clone on a Ventana automated platform and quantified using the QuPath analysis platform, is significantly associated with better clinical outcome in TNBC treated with current standard of care chemotherapy. This observation was confirmed using a second TMA in which a large proportion of the TNBC cases were replicated. When immune topography was taken into account, tumour rich cores showed the greatest predictive power with analysis of PD-L1 expression regardless of cell type (epithelial or lymphoid) demonstrating the most clinical relevance. Looking at breast cancer as a whole, there was also an association between high PD-L1 expression and improved clinical outcome. Moreover, we show for the first time that PD-L1 expression significantly predicts clinical outcome in all ER negative patients, including HER2 enriched disease.

This study highlights the potential risk of obtaining varied results when investigating the role of PD-L1 as a biomarker and may explain some of the inconsistencies within the field. As previously mentioned, there is a range of commercially available antibodies for PD-L1 IHC studies. Mahoney et al. have shown that the region of PD-L1 against which the antibody is raised can have a significant impact on the staining patterns observed [47]. They conclude that antibodies raised against the cytoplasmic domain of PD-L1 can clearly identify the membrane of PD-L1 positive tumour epithelial cells and allow more accurate scoring. Both the Cell Signalling and the Ventana SP142 antibodies are raised against this region while the Abcam antibody is raised against the extracellular domain. Our results suggest that the combination of cytoplasmic domain specific antibodies and their analysis with a validated automated platform, as epitomised by our results with the SP142 antibody, is the best vehicle for IHC detection of PD-L1 in breast FFPE tissue. This is consistent with its development as a companion assay to the Roche/Genentech anti-PD-L1 immunotherapy, MPDL3280A, and observed clinical utility in clinical trial samples [46, 48, 56].

Current standard scoring systems for PD-L1 as a companion diagnostic to immunotherapy find clinical significance in percentages of expression above or below 1% [57]. We also show that the use of a 1% threshold for calling positive PD-L1 expression has clinical utility in predicting outcome in breast cancer in the context of standard of care. This emphasises the importance of accurate reproducible scoring that can discriminate low PD-L1 expression but, at the same time, opens an area of diagnostic value (expression above or below 1%) in which it is notoriously difficult to reach interobserver concordance. As shown using the custom cell lines panel, most interantibody variability was observed at the low levels of expression while concordance was seen at extreme positive or negative levels indicating that multiple factors will impact detection of PD-L1 at clinically relevant levels, as encapsulated by the clinical challenges we face, reporting in our experience of routine PD-L1 diagnostic reflex testing [58]. Quantification by image analysis provides the potential to obtain accurate reproducible results at low expression levels. The ability to dynamically identify sensitivity thresholds within QuPath allows users to visually review PD-L1 detection to ensure accurate and robust assessment at these low expression levels.

This sensitivity and specificity has allowed us to elucidate a role for PD-L1 in predicting response in patients within the ER negative HER2 enriched subgroup. To the best of our knowledge, this is the first time the utility of PD-L1 as a biomarker has been demonstrated to predict improved outcome in this subgroup. Along with TNBC, this is a subgroup of unmet clinical need. While use of HER2 targeted agents has had undeniable clinical benefit, response rates are low with best responses rates (~50%) observed in the neo-adjuvant setting with a combination of targeted agents [59, 60]. Furthermore, for those who do respond, resistance is common, meaning overall survival is poor. PD-L1 may serve to inform clinicians which patients are likely to require additional treatment options to achieve better outcomes.

Of interest, high levels of TILs are associated with better response to Trastuzumab in both the neo-adjuvant and adjuvant setting potentially associated with antibody-dependent cell-mediated cytotoxicity (ADCC) mechanism of action of the drug [61]. Furthermore, the combination of Herceptin and PD1/PD-L1 blockade has shown greater tumour regression in mouse models of HER2+ disease [62]. These observations have led to the PANACEA trial investigating the use of PD1 inhibitors in advanced, Trastuzumab resistant HER2-positive breast cancer (NCT02129556). Final safety data and efficacy results presented at ASCO 2018 are positive showing that the trial has met its primary endpoint [63]. Due to the historic nature of the patient cohort used in the current study, only 2 patients received Trastuzumab and so no correlation can be made between PD-L1 and response to the targeted agent in this study. However, these results indicate that PD-L1 may predict response to standard of care treatment and thus provides clinical utility in this setting. Moreover, our data adds to the evidence from the PANACEA trial suggesting that immune evasion is a mechanism of resistance to Trastuzumab as well as standard of care chemotherapy and contributes to disease progression in advanced HER2+ breast cancer.

Muenst et al. [22] previously published that high PD-L1 expression was associated with poor prognosis in breast cancer as a whole. Upon subset analysis, this observation was significant within the Luminal B HER2-, Luminal B HER2+, and HER2 enriched and basal-like subtypes. This is in stark contrast to our findings as well as numerous other findings centred on PD-L1 expression as a positive prognostic factor in TNBC [21, 23, 24]. However, the validity of their results has previously been challenged with particular emphasis on the choice of antibody used in the study [64].

In addition to highlighting the novel role of PD-L1 in predicting outcome in ER-negative breast cancer, this study has also shown that high PD-L1 expression is associated with improved outcome in breast cancer as a whole. This is in contrast to the overall findings from two recent meta-analyses showing high PD-L1 expression associated with poor outcome [65, 66]. However, two studies from within these meta-analyses did also report a significant association between high PD-L1 expression and improved outcome. One study utilised the Abcam antibody, dichotomised patients based on the median from an Allred scoring system and observed a significant association between high PD-L1 expression and OS [67]. The second study examined PD-L1 mRNA utilising the same RNAScope in situ hybridisation assay applied in this study. Schlaper et al. found that PD-L1 expression (any expression above the noise threshold determined by quantification of a negative control) was significantly associated with RFS and the presence of TILs [21]. This highlights again the importance for consistency in how PD-L1 is quantified and thresholds for positivity are determined. It is also important to consider the treatment received by patient cohorts. All patients within the NIB Breast cohort received adjuvant anthracycline based chemotherapy. While all molecular subgroups of breast cancer are represented, the cohort does not include lower risk patients who are treated without systemic chemotherapy. Therefore, the positive prognostic role of PD-L1 may only be apparent in the context of chemotherapy.

The finding that PD-L1 expression, regardless of the cell type (epithelial or lymphoid), is prognostic is keeping with the guidelines for the companion assay for Atezolizumab using SP142 [68]. Furthermore, the strong correlation between epithelial and stromal PD-L1 expression indicates that expression driven by an adaptive immune resistance may be of most clinical relevance in predicting outcome in breast cancer. This is supported further by the significant association between PD-L1 expression and an “anti-tumour” Th1/M1 polarised tumour microenvironment which we have previously shown to be significantly associated with improved survival in the context of SoC chemotherapy in TNBC [9]. This PD-L1 expression is likely driven by the presence of DNA repair defects [69] which also underpins the favourable response to DNA-damaging chemotherapies and indicates that the cohort of patients that gain clinical benefit from the current standard of care chemotherapy are likely to be the same patients that will respond to PD1/PD-L1 blockade. While significant, the correlation between PD-L1 and percentage of stromal TILs was observed to be weak with high TILs observed in cases with low PD-L1 expression. This further emphasises the complexities of the interaction between the tumour and the host immune system and highlights the continuous need for alternate treatment options to be identified to improve outcome in patients who do not display adaptive immune resistance.

## 5. Conclusion

This study highlights the need for high quality information at multiple levels (e.g., quantification, knowledge of immune topography/contexture, and clinical annotation) in order to accurately interpret the utility of a biomarker. We have shown the impact of high-resolution image analysis on PD-L1 interpretation from antibody choice to IHC versus mRNA-based quantification. This comprehensive comparison would not have been possible without the objective, reproducible and accurate PD-L1 quantification provided by our digital pathology platform QuPath. We believe this study provides vital information impacting on the clinical delivery and interpretation of PD-L1 as a biomarker and provides clarity to the role of PD-L1 in the prediction of response to standard of care in breast cancer.

## Data Availability

All data related to the clinical cohorts and TMAs are available upon application to the Northern Ireland Biobank.

## Supplementary Materials

Supplementary Materials Supplementary Figure 1. (A) Overview of tissue microarray design from the cores chosen in Figure 2A with each column representing an individual patient. Where the size of the tumour was insufficient, cores targeting enriched epithelium were prioritized as denoted with *∗*. (B) Kaplan Meier Plots of overall survival stratified based on PD-L1 expression above or below 1% as determined by the (i) SP142 or (ii) by RNAScope. (C) Images (x5) from whole face sections of TNBC stained with PD-L1, adjacent to where tumour rich TMA cores were taken. (i) - No expression, (ii) - Low level of expression on tumour epithelium*∗* but also on surrounding stromal cells, (iii) - High expression on tumour epithelium. Inlays x15 magnification. Supp Figure 2. (A) Kaplan Meier Plots of overall survival stratified based on PD-L1 expression above or below 1% in the three different core types (i)TS, (ii)T and (iii) TL. (B) Kaplan Meier Plots of overall survival stratified based on (i) tumour or (ii) stroma derived PD-L1 expression above or below 1%. (C) Kaplan Meier plots of (i) relapse free and (ii) overall survival stratified based on percentage stromal TIL expression in T cores above or below 30%. Supp Figure 3. Kaplan Meier Plots of overall survival stratified based on PD-L1 expression above or below 1% in (A) breast cancer as a whole, (B) TNBC, (C) Luminal A, (D) Luminal B/HER2 negative, (E) Luminal B/HER2 positive, (F) HER2 positive and (G) ER negative breast cancer. Supplementary Tables. Supp Table 1: Clinical and Pathological Characteristics of (A) 90 patients included in bespoke triple negative TMA, and (B) the Breast 300 cohort. Supp Table 2: Antibody conditions for IHC. Supp Table3: Lin's concordance coefficients and 95% Confidence intervals for correlation between PD-L1 expression using three different antibodies and two different automated staining platforms. Supp Table 4-6. Full details of Hazard ratios, 95% confidence intervals and p-values for all survival analysis.
