# Supplementary material for: Automated Tumour Recognition and Digital Pathology Scoring Unravels New Role for PD-L1 in Predicting Good Outcome in ER-/HER2+ Breast Cancer
Source: J Oncol. 2018 Dec 17;2018:2937012. doi: 10.1155/2018/2937012 (PMC6311859; doi:10.1155/2018/2937012)
Supplement: Supplementary Materials — Supplementary Figure 1. (A) Overview of tissue microarray design from the cores chosen in Figure 2A with each column representing an individual patient. Where the size of the tumour was insufficient, cores targeting enriched epithelium were prioritized as denoted with ∗. (B) Kaplan Meier Plots of overall survival stratified based on PD-L1 expression above or below 1% as determined by the (i) SP142 or (ii) by RNAScope. (C) Images (x5) from whole face sections of TNBC stained with PD-L1, adjacent to where tumour rich TMA cores were taken. (i) - No expression, (ii) - Low level of expression on tumour epithelium∗ but also on surrounding stromal cells, (iii) - High expression on tumour epithelium. Inlays x15 magnification. Supp Figure 2. (A) Kaplan Meier Plots of overall survival stratified based on PD-L1 expression above or below 1% in the three different core types (i)TS, (ii)T and (iii) TL. (B) Kaplan Meier Plots of overall survival stratified based on (i) tumour or (ii) stroma derived PD-L1 expression above or below 1%. (C) Kaplan Meier plots of (i) relapse free and (ii) overall survival stratified based on percentage stromal TIL expression in T cores above or below 30%. Supp Figure 3. Kaplan Meier Plots of overall survival stratified based on PD-L1 expression above or below 1% in (A) breast cancer as a whole, (B) TNBC, (C) Luminal A, (D) Luminal B/HER2 negative, (E) Luminal B/HER2 positive, (F) HER2 positive and (G) ER negative breast cancer. Supplementary Tables. Supp Table 1: Clinical and Pathological Characteristics of (A) 90 patients included in bespoke triple negative TMA, and (B) the Breast 300 cohort. Supp Table 2: Antibody conditions for IHC. Supp Table3: Lin's concordance coefficients and 95% Confidence intervals for correlation between PD-L1 expression using three different antibodies and two different automated staining platforms. Supp Table 4-6. Full details of Hazard ratios, 95% confidence intervals and p-values for all survival analysis. [file 2937012.f1.pdf]

Supp Figure 1

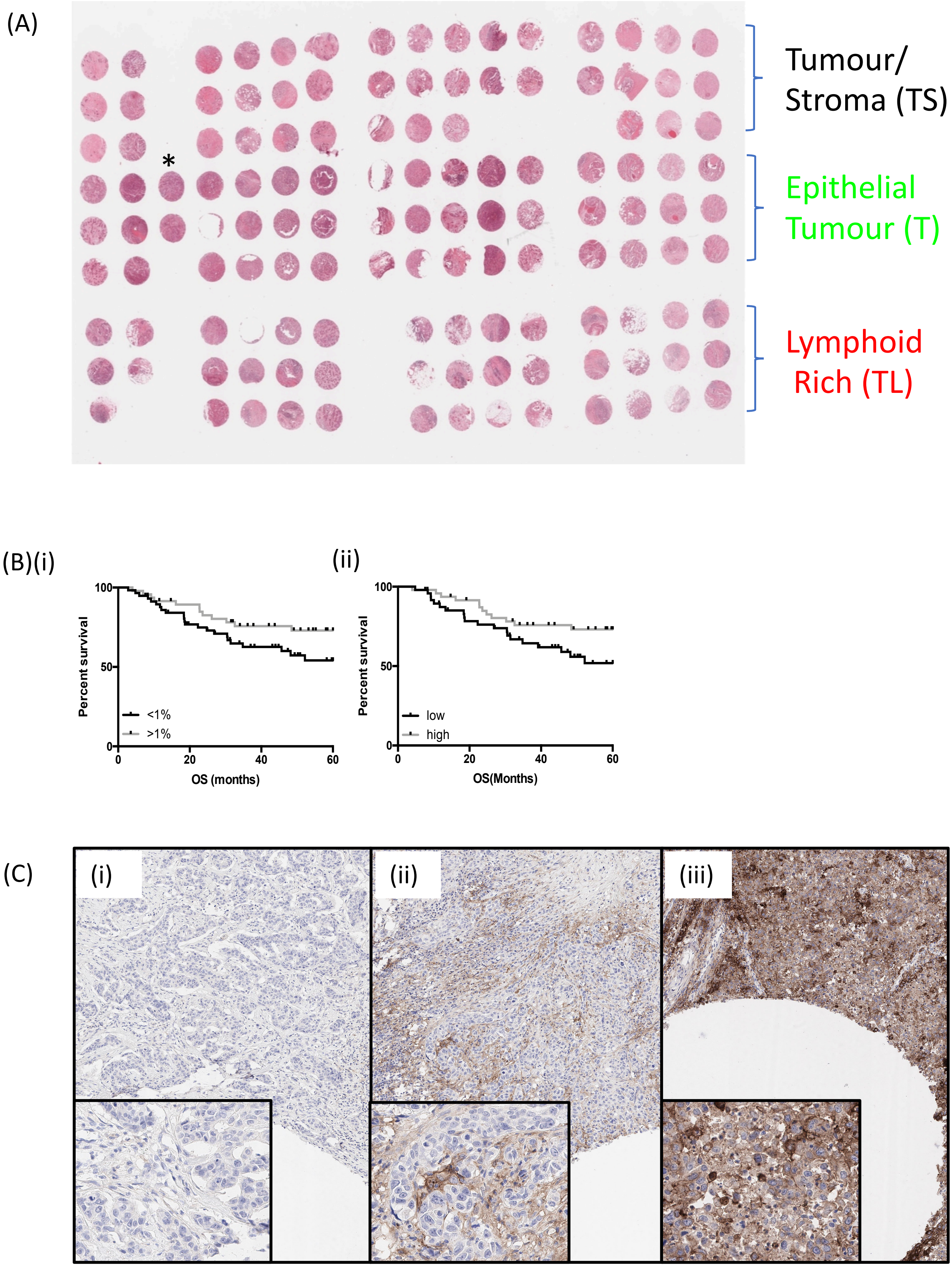

Supp Figure 2

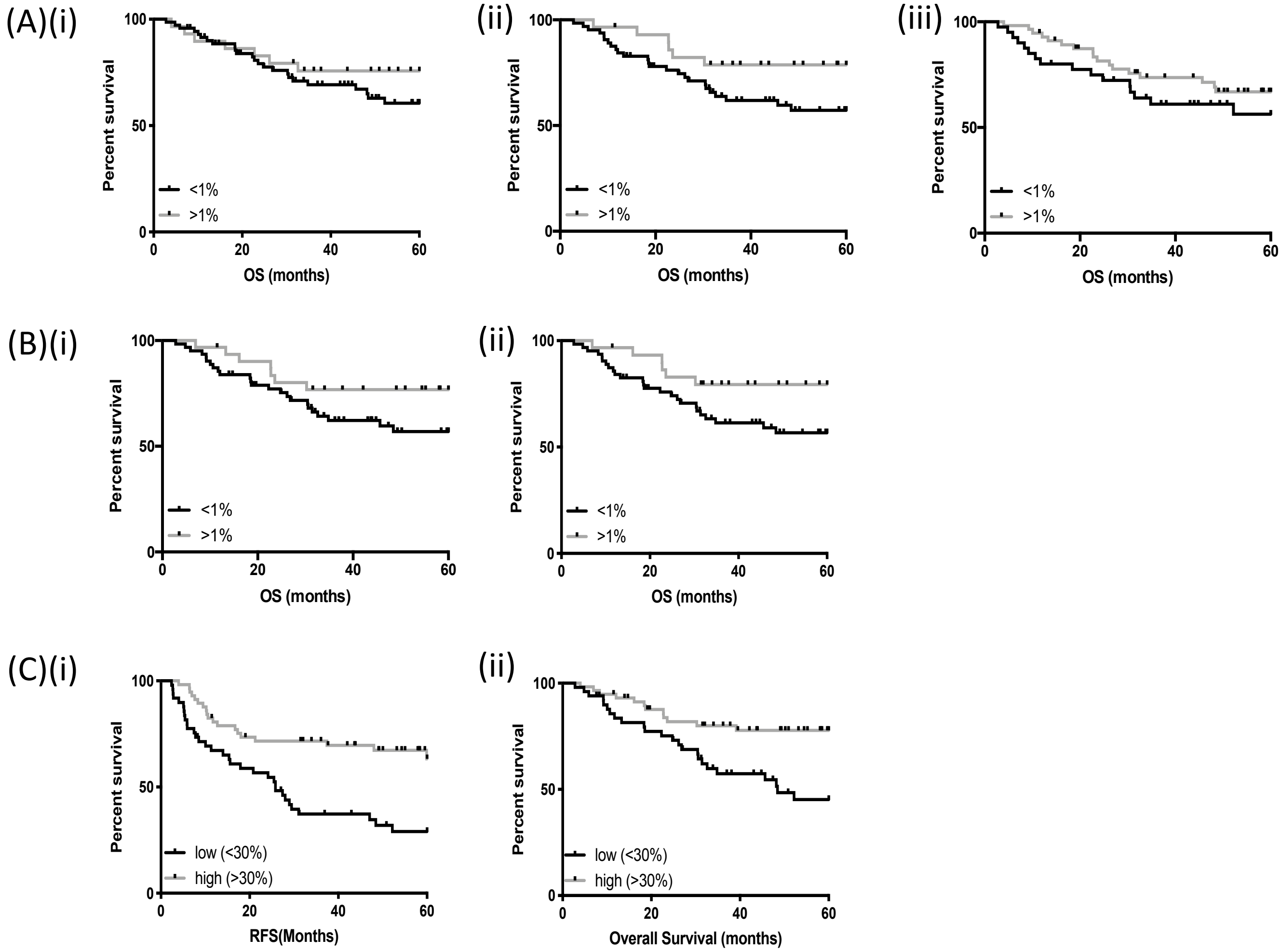

Supplementary Figure 3

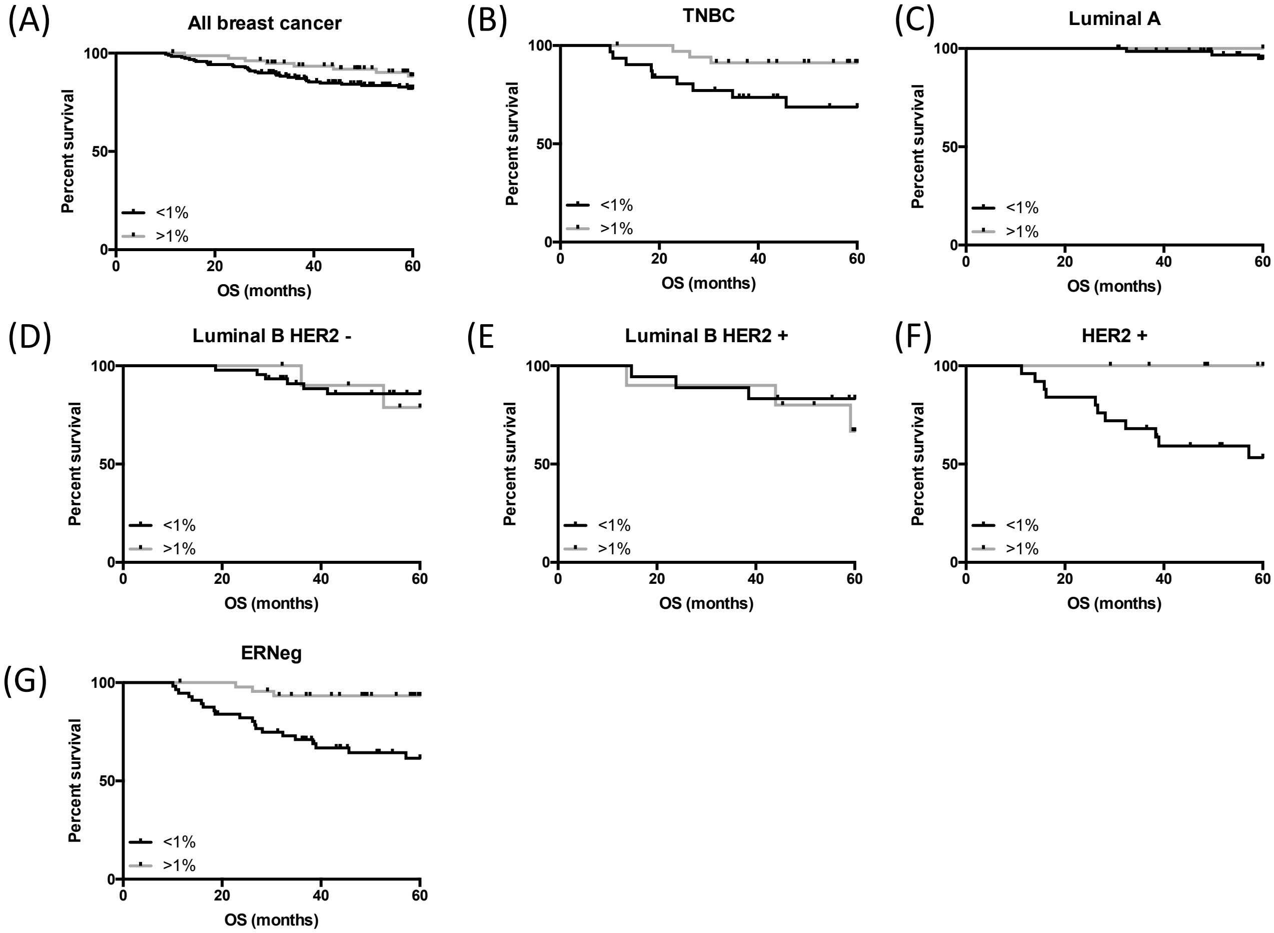

Supp Table 1:
(a) 9 core TNBC TMA

Variable (N=109)	N (%)
Age (mean (range))	56.69 (28-90)
Grade N(%)	
G2	13 (11.9)
G3	96 (88.1)
T code N(%)	
T1	31 (28.5)
T2	53 (48.6)
T3	19 (17.4)
T4	6 (5.5)
N code N(%)	
N0	63 (57.8)
N1	29 (26.6)
N2	8 (7.35)
N3	8 (7.35)
Unknown	1 (0.9)
Chemotherapy	
None	18 (16.5)
FEC	81 (74.3)
FEC-D	9 (8.3)
AC	1 (0.9)

(b) 300 breast cohort

Variable (*N* = 293)	*N (%)*
Tumour grade	
G1	5 (2)
G2	119 (41)
G3	169 (58)
pN stage (nodal involvement)	
N0	127 (43)
N1mi	4 (1)
N1	95 (32)
N2	38 (13)
N3	29 (10)
pT stage	
T1	65 (22)
T2	181 (62)
T3	41 (14)
T4	6 (2)
Histological type	
Ductal (no special type)	230 (78)
Lobular (no special type)	31 (11)
Mixed ductal and lobular	25 (9)
Other	7 (2)
ER status (at diagnosis)	
Positive	175 (60)
Negative	115 (39)
Unknown	3 (1)
PR status (at diagnosis)	
Positive	115 (39)
Negative	139 (47)
Unknown	39 (13)
HER2 status (at diagnosis)	
Positive	75 (26)
Negative	192 (66)
Unknown	26 (9)

Supp Table 2:

Antibody	Company/Clone	Platform/pre-treatment/dilution	Detection chemistry	Mono-/polyclonal	Epitope
ER	Ventana/SP1	BenchMark/CC1 60mins	Ultraview	Rabbit Monoclonal	synthetic peptide corresponding to the C-terminal
PR	Ventana/1E2	BenchMark/CC1 60mins	Ultraview	Rabbit Monoclonal	Synthetic peptide identified as an area of potential high antigenicity common to progesterone receptor A and B forms
HER2	Ventana/4B5	BenchMark/CC1 30mins	Ultraview	Rabbit Monoclonal	Internal domain of HER2
PDL1	Ventana /SP142 (originally sourced from Spring Bioscience)	BenchMark/CC1 56mins/1:40	Optiview with amplification	Rabbit Monoclonal	Synthetic peptide derived from C-terminus
PDL1	Abcam/28-8	BenchMark/CC1 56mins/1:800	Optiview with amplification	Rabbit Monoclonal	extracellular domain of PDL1 (Phe19-Thr239)
PDL1	Cell Signalling /405.9A11	BenchMark/CC1 56mins/1:20	Optiview with amplification	Mouse Monoclonal	Synthetic peptide derived from C-terminus

Supp Table 3:

	PDL1 Abcam Benchmark	PDL1 Abcam Bondmax	PDL1 SP142 Benchmark	PDL1 SP142 Bondmax	PDL1 Cell Signalling Benchmark	PDL1 Cell Signalling Bondmax
PDL1 Abcam Benchmark		0.7707 (95%CI 0.4066-0.9233)	0.8254 (95%CI 0.5775-0.9541)	0.8285 95%CI 0.5215-0.9456	0.7718 (95% CI 0.4089 – 0.9239)	0.8571 (95%CI 0.5843 – 0.9558)
PDL1 Abcam Bondmax	0.7707 (95%CI 0.4066-0.9233)		0.9826 95%CI 0.9407-0.0049	0.9908 95%CI 0.9683-0.9973	0.9992 (95%CI 0.9971-0.9997)	0.9774 95%CI 0.9238-0.9934
PDL1 SP142 Benchmark	0.8254 (95%CI 0.5775-0.9541)	0.9826 95%CI 0.9407-0.0049		0.9973 95%CI 0.9905-0.9992)	0.9823 95%CI 0.9501-0.9949	0.9985 95%CI 0.9947-0.9995
PDL1 SP142 Bondmax	0.8285 95%CI 0.5215-0.9456	0.9908 95%CI 0.9683-0.9973	0.9973 95%CI 0.9905-0.9992)		0.9908 95%CI 0.9680-0.9974	0.9961 95%CI 0.9864-0.9989
PDL1 Cell Signalling Benchmark	0.7718 (95% CI 0.4089 – 0.9239)	0.9992 (95%CI 0.9971-0.9997)	0.9823 95%CI 0.9501-0.9949	0.9908 95%CI 0.9680-0.9974		0.9772 95% CI 0.9226-0.9934)
PDL1 Cell Signalling Bondmax	0.8571 (95%CI 0.5843 – 0.9558)	0.9774 95%CI 0.9238-0.9934	0.9985 95%CI 0.9947-0.9995	0.9961 95%CI 0.9864-0.9989	0.9772 95% CI 0.9226-0.9934)	

Supp Table 4a

Relapse Free Survival			Univariate		
All core types - Overall core score			HR	95% CI	p-value
SP142		N(n)104 (52)			
PDL1	>1%	46 (18)	1		
	<1%	58 (34)	0.536	0.316-0.94	0.0294*
RNAScope		N (n) 96 (45)			
PDL1	high	48 (19)	1		
	low	48 (26)	0.632	0.352- 1.136	0.1258

Supp Table 4b

Overall Survival			Univariate		
All core types - Overall core score			HR	95% CI	p-value
SP142		N(n)104 ()			
PDL1	>1%	46 (12)	1		
	<1%	58 (23)	0.548	0.288- 1.084	0.0858
RNAScope		N (n) 96 (32)			
PDL1	high	48 (12)	1		
	low	48 (20)	0.519	0.260-1.045	0.0669

Supp Table 5

Relapse Free Survival			Univariate		
9 core TMA SP142		N(n)	HR	95% CI	p-value
TS Cores		100 (47)			
PDL1	>1%	29 (11)	1		
	<1%	71 (38)	0.640	0.360-1.225	0.1914
T cores		93 (45)			
PDL1	>1%	29 (9)	1		
	<1%	64 (36)	0.445	0.271-0.917	0.0255*
TL cores		96 (49)			
PDL1	>1%	56 (24)	1		
	<1%	40 (25)	0.562	0.305-0.972	0.0405*
T cores tumour region		93 (45)			
PDL1	>1%	31 (10)	1		
	<1%	62 (35)	0.455	0.274-0.909	0.0237*
T cores stroma region		93 (45)			
PDL1	>1%	30 (9)	1		
	<1%	63 (36)	0.421	0.260-0.872	0.0164*

Supp Table 5b

Overall Survival			Univariate		
9 core TMA SP142		N(n)	HR	95% CI	p-value
TS Cores		100(31)			
PDL1	>1%	29 (7)	1		
	<1%	71 (24)	0.631	0.308-1.402	0.2785
T cores		93 (31)			
PDL1	>1%	29 (6)	1		
	<1%	64 (25)	0.457	0.244-1.074	0.0767
TL cores		96 (33)			
PDL1	>1%	56 (17)	1		
	<1%	40 (16)	0.668	0.326-1.326	0.2426
T cores tumour region		93 (31)			
PDL1	>1%	31 (7)	1		
	<1%	62 (24)	0.505	0.262-1.133	0.1040
T cores stroma region		93 (31)			
PDL1	>1%	30 (6)	1		
	<1%	63 (25)	0.434	0.236-1.026	0.0586

Supp Table 5b

			Univariate		
Relapse Free Survival		N(n)	HR	95% CI	p-value
TILs	>30%	57 (19)	1		
	<30%	49 (33)	0.3787	0.2173-0.66	0.0004
Overall Survival		N(n)	HR	95% CI	p-value
TILs	>30%	58 (23)	1		
	<30%	50 (24)	0.3712	0.192-0.719	0.0034

Supp Table 6a

Relapse Free Survival			Univariate		
Breast 300 TMA SP142		N(n)	HR	95% CI	p-value
All breast		268 (65)			
PDL1	>1%	78 (13)	1		
	<1%	190 (53)	0.547	0.350-0.994	0.0477*
Luminal A		78 (10)			
PDL1		10 (0)	1		
		68 (10)	0.314†	0.051-1.928	0.2112
Luminal B HER2-		66 (14)			
PDL1	>1%	11 (2)	1		
	<1%	55 (12)	0.620	0.183-2.381	0.5272
Luminal B HER2 +		28 (9)			
PDL1	>1%	10 (2)	1		
	<1%	18 (7)	0.484	0.136-2.041	0.3550
HER2 +		36 (12)			
PDL1	>1%	11 (2)	1		
	<1%	25 (10)	0.350	0.131-1.378	0.1553
TNBC		67 (21)			
PDL1	>1%	35 (7)	1		
	<1%	32 (14)	0.355	0.148-0.841	0.0190*
ER -		103 (33)			
PDL1	>1%	46 (9)	1		
	<1%	57 (24)	0.360	0.195-0.761	0.0063**

† Mantel-Haenszel HR reported

Supp Table 6b

Overall Survival			Univariate		
Breast 300 TMA SP142		N(n)	HR	95% CI	p-value
All breast		268 (40)			
PDL1	>1%	78 (8)	1		
	<1%	190 (32)	0.584	0.316-1.224	0.1695
Luminal A		78 (3)			
PDL1		10 (0)	1		
		68 (3)	0.314†	0.0115-8.576	0.4714
Luminal B HER2-		66 (8)			
PDL1	>1%	11 (2)	1		
	<1%	55 (6)	1.297	0.237-7.38	0.7493
Luminal B HER2 +		28 (6)			
PDL1	>1%	10 (3)	1		
	<1%	18 (3)	1.85	0.360-10.30	0.4437
HER2 +		36 (11)			
PDL1	>1%	11 (0)	1		
	<1%	25 (11)	0.215†	0.062-0.745	0.0153*
TNBC		67 (12)			
PDL1	>1%	35 (3)	1		
	<1%	32 (9)	0.255	0.087-0.861	0.0269*
ER -		103 (23)			
PDL1	>1%	46 (3)	1		
	<1%	57 (20)	0.154	0.103-0.526	0.0005***

† Mantel-Haenszel HR reported

Supp Table 6c

Relapse Free Survival			Multivariate- Stepwise regression		
ER Neg SP142		N(n)103 (33)	HR	95% CI	p-value
PDL1	>1%	57 (24)	1		
	<1%	46 (9)	0.386	0.179-0.833	0.0153*
Lymph node	no	54 (10)	1		
	yes	49 (23)	0.308	0.146-0.651	0.0020**

Supp Table 6d

Overall Survival			Multivariate- Stepwise Regression		
ER Neg SP142		N(n)103 (23)	HR	95% CI	p-value
PDL1	>1%	57 (20)	1		
	<1%	46 (3)	0.185	0.055-0.942	0.00656**
Lymph node	no	54 (4)	1		
	yes	49 (19)	0.189	0.064-0.56	0.00263**
